# Climate-smart agriculture practices influence weed density and diversity in cereal-based agri-food systems of western Indo-Gangetic plains

**DOI:** 10.1038/s41598-021-95445-1

**Published:** 2021-08-05

**Authors:** Hanuman S. Jat, Virender Kumar, Suresh K. Kakraliya, Ahmed M. Abdallah, Ashim Datta, Madhu Choudhary, Mahesh K. Gathala, Andrew J. McDonald, Mangi L. Jat, Parbodh C. Sharma

**Affiliations:** 1 ICAR-Central Soil Salinity Research Institute (CSSRI), Karnal, India; 2 International Rice Research Institute (IRRI), Los Banos, Philippines; 3 Faculty of Agriculture, Damanhour University, Damanhour, Egypt; 4 International Maize and Wheat Improvement Center (CIMMYT), Dhaka, Bangladesh; 5 Collage of Agriculture and Life Sciences, Cornell University, Ithaca, NY USA; 6 International Maize and Wheat Improvement Center (CIMMYT), New Delhi, India

**Keywords:** Ecology, Plant sciences, Environmental sciences

## Abstract

Climate-smart agriculture (CSA)-based management practices are getting popular across South-Asia as an alternative to the conventional system for particular weed suppression, resources conservation and environmental quality. An 8-year study (2012–2013 to 2019–2020) was conducted to understand the shift in weed density and diversity under different CSA-based management practices called scenarios (Sc). These Sc involved: Sc1, conventional tillage (CT)-based rice–wheat system with flood irrigation (farmers’ practice); Sc2, CT-rice, zero tillage (ZT)-wheat–mungbean with flood irrigation (partial CA-based); Sc3, ZT rice–wheat–mungbean with flood irrigation (partial CSA-based rice); Sc4, ZT maize–wheat–mungbean with flood irrigation (partial CSA-based maize); Sc5, ZT rice–wheat–mungbean with subsurface drip irrigation (full CSA-based rice); and Sc6, ZT maize–wheat–mungbean with subsurface drip irrigation (full CSA-based maize). The most abundant weed species were *P. minor* > *A. arvensis* > *M. indicus* > *C. album* and were favored by farmers’ practice. However, CSA-based management practices suppressed these species and favored *S. nigrum* and *R. dentatus* and the effect of CSAPs was more evident in the long-term. Maximum total weed density was observed for Sc1, while minimum value was recorded under full CSA-based maize systems, where seven weed-species vanished, and *P. minor* density declined to 0.33 instead of 25.93 plant m^−2^ after 8-years of continuous cultivation. Full CSA-based maize–wheat system could be a promising alternative for the conveniently managed rice–wheat system in weed suppression in north-west India.

## Introduction

Rice (*Oryza sativa *L.)—wheat (*Triticum aestivum *L.) (RW) is the major agri-food system occupying an area of 13.5 Mha in the Indo-Gangetic Plains (IGP) of South Asia; of which ten Mha in India, with almost 50% (5 Mha) are in western IGP comprising of Punjab, Haryana and western Uttar Pradesh, the food basket of India^1,2^. With conventional management practices, high productivity derived from this continuous RW system are at the cost of over-exploitation of resources (i.e. groundwater, soil, energy) and high use of inputs, (i.e. irrigation, fertilizer, herbicides and pesticides)^3–6^. The sustainability of the RW system in western Indo-Gangetic plains (IGP) is doubtful due to the rapid decline in soil and water resources, and environmental quality^7^. Furthermore, continued cultivation of the same cropping system (i.e. rice–wheat system) over the last five decades allowed certain weed species like *Phalaris minor* to adapt, increase their establishment, seedbank and profusion^8^. Such weeds adversely affect resource use efficiency (light, water and nutrients), and crop productivity^3,9^. In the western IGP, weed infestation negatively influences wheat production, in particular under conventional wheat management systems^10^. Weeds infestation, could decrease wheat yield by 50–80%^11^ or even 100%^3^ in South Asian IGP. On a global scale, the yield losses from weeds amounts to 40%^12^, which is more than those coming from collective effect of diseases, insect and pests^13^. Under the RW cropping system *Phalaris minor* is the most predominant weed, due to its ability to survive under the anaerobic conditions (created during rice cultivation)^10,14,15^. Furthermore, *P. minor* is gaining resistance against numerous common herbicides^10,15^. Besides its adverse impacts on the environment and its high cost, presently herbicide resistance is emerging at large scale area in different crops and cropping systems^16^, making chemical control less reliable^17^. Therefore, weed management is becoming increasingly important in future cropping systems^18^.

The density, diversity and composition of weed species depend on the nature of the crop management strategies which may be indicative for weed management solutions^19^. On a global scale, most weed investigations have been dedicated to the study of crop-weed competition responses to tillage practices or weed communities alone^17,20^. Under conventional agriculture, tillage practices change the weed seed distributions both vertically and horizontally, leading to seedbank deeper in the soil, thus affect seeds viability, emergence and seedlings’ survival, and modifies weed species abundance and composition^9,21–23^. However, conservation agriculture (CA) comprising of zero tillage (ZT), crop residue retention and crop diversification influence the weed density and biomass much compared to conventional-till (CT) management practices. In ZT, a higher portion of weed seeds presents close to the soil surface, increasing the potential of its germination during the initial years^24^. However, in ZT, weed seeds are more vulnerable to predation, and mortality and lose viability risks, leading to unviable seeds and seedbank depletion over time^1,21,25,26^. Thus, the adoption of ZT resulted in seedbank tiredness and reduces the aboveground weed infestation^25^. Moreover, retaining crop residue on the soil surface imposes a physical barrier to emerging seeds and restricts the growth of germinated ones by limiting light availability^21,27^. Crop residue might also suppress weeds germination due to releasing biochemicals (allelopathy), which tend to affect smaller seeds, in particular^28^. In ZT, retaining rice residue of 5.0 Mg ha^−1^ decreased the weed dry biomass in wheat by 23.4–30.3%^10^ and weed density by 22–76%^29^. It is rich-established that the continuous cropping RW system increases weed species adaption and intensification^25,30^. Diversifying crop rotation by adding crop species sown in varied dates, having dissimilar life cycles and associated managements may suppress weeds^9,21,30,31^. Increasing rotational diversity reduces weed density by 65% under ZT and 41% under CT^30^. Sustainable intensification through replacing of crop or addition of extra leguminous crop in RW system found to be an efficient approach for suppressing annual weeds like *P. minor* and *A. arvensis*^23,25,28,32^. Interestingly, using different crops increases predation pressure^33^ and promotes the predation of diverse weed seeds^34^.

Although weeds are a threat to crops, it is an integral component in the Agri-ecosystem and could be considered as a bio-indicator for adopted management practices in a particular region^19^. Since applying CA-based management practices within the Agri-ecosystem might shift weed density and diversity^25,26^, thus, only weed species that can adapt to certain crop and management practices will grow and proliferate, while others will disappear. In this way, the emerged weeds might be considered as bio-indicators for applied management practices. However, due to the negative impression and its impacts on crop production, weeds as bio-indicators in particular Agri-ecosystem/region were poorly understood and have found little interest.

Climate-smart agriculture (CSA)-based management practices are evolving as a viable and sustainable alternative to conventional RW cropping systems across south Asia for better resources conservation and yield stability^35^. CSA-depends on agronomic aspects for improving systems adaptive capacity, sustaining food security while reducing the environmental footprints and can influence weed density and diversity composition in particular agro-ecosystems^36^. CSA-based management practices rely on a wide range of indicators, i.e. tillage, crop establishment, crop residue retention, crop diversification, and precise water and nutrient management, along with the use of information and communication tools (ICTs) for timely implementation of crop practices^30,37^. Keeping the soil mulch around the year (crop residue retention) and layered with precise water application through subsurface drip irrigation (SDI) helped in more predation and less germination of weeds seeds due to less sunlight and moisture on the near the soil surface^4,38^. Besides the well-known benefits of the SDI system, i.e. minimizing evaporation from the soil, efficient water, and fertilizers application and use and reducing labor cost, the SDI system also discourages weed germination and growth^39^. However, most of the research done was conducted with one or two treatment bases like tillage and mulch on the weed community composition, particularly in the wheat-based system. Research investigations on the assessment of weed community responses to CSA are inadequately understood, which is vital for integrated weed management in the wheat-based cropping systems. Long-term field trials based on CSA practices are required to understand the changes in plant community diversity and composition and could give insight into long-term effects and best ecological indicators “weed species” for different management practices. We hypothesized that CSA management scenarios (layering of different indicators/practices) could reduce weed density and composition, and diversity indices with no negative impact on wheat performance under rice/maize-based systems.

## Results

### Weed density

**Figure 1:**
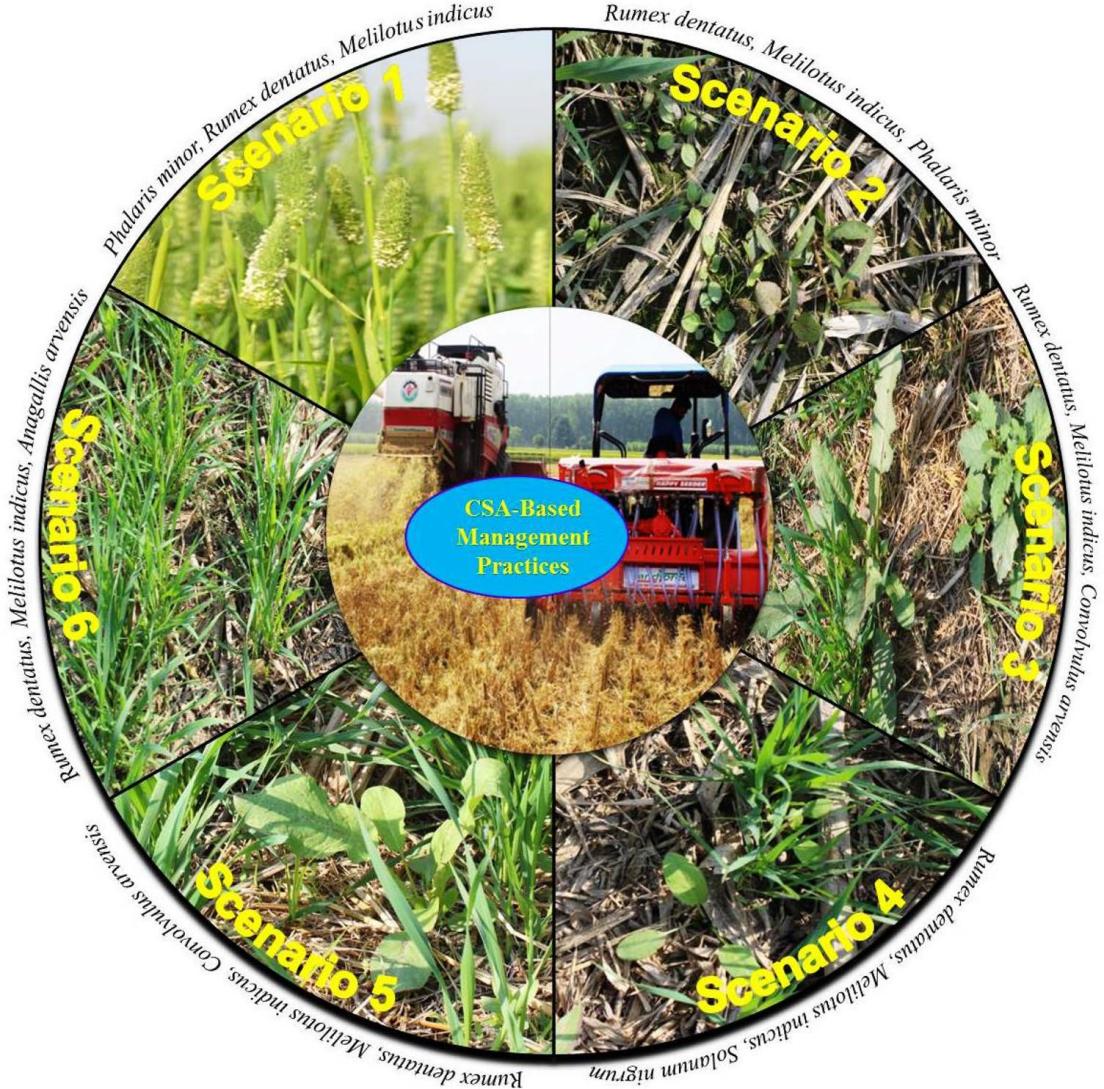
Schematic diagram of dominant weed species under climate-smart agriculture (CSA)-based management scenarios. *Sc1* conventional rice–wheat system with flood irrigation (FI), *Sc2* conservation agriculture (CA)-based rice–wheat–mungbean system with FI, *Sc3* partial CSA-based rice–wheat–mungbean system with FI, *Sc4* partial CSA-based maize–wheat–mungbean system with FI. *Sc5* full CSA-based rice–wheat–mungbean system with subsurface drip irrigation (SDI), *Sc6* full CSA-based maize–wheat–mungbean system with SDI. The figure has been generate using Microsoft PowerPoint office professional Plus (2010), version (14.0.4734.1000).

**Figure 2:**
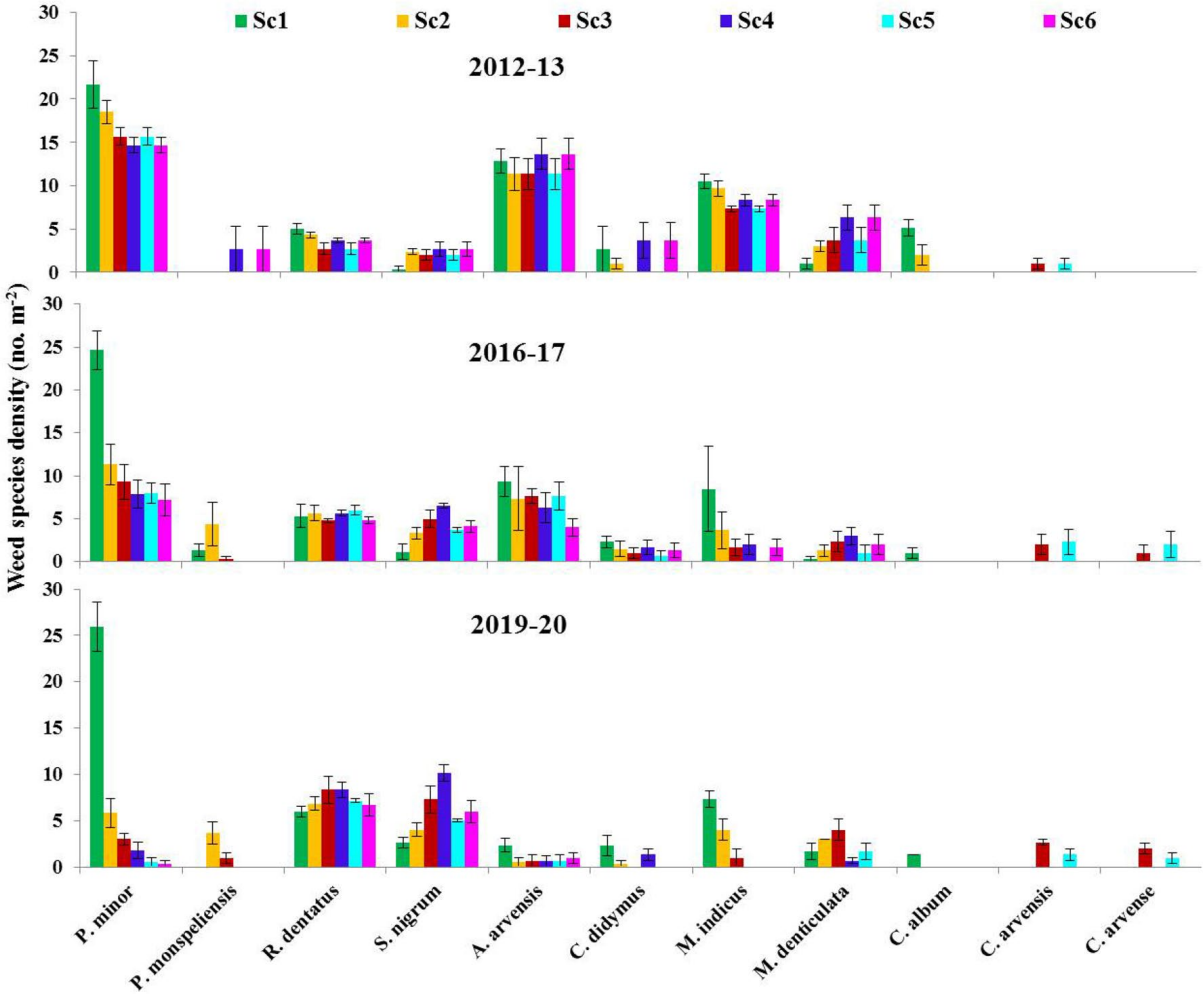
Effects of climate-smart agriculture-based management practices on major weed species density (no. m^−2^) at 45 days after planting during different years. Vertical bars indicate ± SE of mean. *Sc1* conventional rice–wheat system with flood irrigation (FI), *Sc2* conservation agriculture (CA)-based rice–wheat–mungbean system with FI, *Sc3* partial CSA-based rice–wheat–mungbean system with FI, *Sc4* partial CSA-based maize–wheat–mungbean system with FI, *Sc5* full CSA-based rice–wheat–mungbean system with subsurface drip irrigation (SDI), *Sc6* full CSA-based maize–wheat–mungbean system with SDI. The figure has been generate using Microsoft Excell office professional Plus (2010), version (14.0.4734.1000).

In the current study, eleven species were observed, three of which are perennials species (Table S1). Weed density was highly affected by CA/CSA-based management practices (scenarios) across the years. The details of the tested six scenarios are given in Table 1. Weed species, i.e., *Phalaris minor, Anagallis arvensis, Rumex dentatus, Solanum nigrum, Melilotus indicus,* and *Chenopodium album,* were the most abundant (Figs. 1, 2). Under farmer’s practice (Sc1), all weed species almost exhibited the same density across years except for *A. arvensis, M. indicus* and *C. album* in which their density was reduced over time (Fig. 2). The density of *P. minor* was highest (25.9 plants m^−2^) under Sc1 and followed by Sc2 (5.8 plants m^−2^) and was negligible under CSA-based systems (Figs. 1, 2). Farmer’s practice (Sc1), favored *P. minor, R. dentatus, A. arvensis, M. indicus,* and *C. album*, while CSA-based management practices (Sc3–6) suppressed the density of these species, except *R. dentatus*. However, all CSA/CA- scenarios increased the density of *S. nigrum and M. denticulata*. Moreover, two perennials weed species (*C. arvensis* and *C. arvense*) emerged only under CSA-based rice systems (Sc3 and Sc5). After 8 years, *S. nigrum and R. dentatus* recorded the highest density for all CA/CSA scenarios.

**Table 1:** Drivers of agricultural change, crop rotation, tillage, crop establishment method, and residue and water management under different scenarios.

Scenario	Drivers of change	Crop rotations	Tillage	Crop establishment method	Residue management	Water management
Sc1	Business as usual (Farmer’s Practice)	Rice–wheat—fallow	CT–CT	Rice: transplanting Wheat: broadcast	All residue removed	Flood irrigation in all crops
Sc2	Increase food production, income and nutrition through intensification and best management practices	Rice–wheat–mungbean	CT–ZT–ZT	Rice: transplanting Wheat: drill seeding Mungbean: drill/relay	Full (100%) rice and anchored wheat residue retained on the soil surface; full mungbean residue incorporated	Same as in scenario 1
Sc3	Deal with the rising scarcity of labor, water, energy, degrading soil health, and emerging climatic variability	Rice–wheat–mungbean	ZT–ZT–ZT	Rice: drill seeding Wheat: drill seeding Mungbean: drill/relay	Full (100%) rice and mungbean; anchored wheat residue retained on the soil surface	Same as in scenario 1
Sc4	Futuristic cropping system to deal with same issues as in Sc3	Maize–wheat–mungbean	ZT–ZT–ZT	Maize: drill seeding Wheat: drill seeding Mungbean: drill/relay	Maize (65%) and full mungbean; anchored wheat residue retained on the soil surface	Same as in scenario 1
Sc5	SI of RW system with precise water and N-management to deal with same issues as in Sc3	Rice–wheat–mungbean	ZT–ZT–ZT	Same as in Sc3	Same as in Sc3	Subsurface drip irrigation (SDI)
Sc6	SI of MW systems with precise water and N-management to deal same issues as in Sc3	Maize–wheat–mungbean	ZT–ZT–ZT	Same as in Sc4	Same as in Sc3	Same as in scenario 5

*CT* conventional tillage, *ZT* zero till, *CA* conservation agriculture, *SI* sustainable intensification, *RW* rice–wheat, *MW* maize–wheat, *SDI* subsurface drip irrigation.

The effects of CSA-based management practices were greatest on two abounded species, in which all CSA-scenarios, once adopted) after 1 year) the emergence of *C. album* was eliminated, meanwhile, the density of *M. indicus* was gradually reduced to be zero under Sc4–6, after 8 years of conversion to CSA (Fig. 2)*.* In Sc4, the density of *P. minor* was lowered down by 32.3, 68.0 and 93.17%, after 1, 4 and 8 years, respectively, than farmers’ practice (Figs. 1, 2). In Sc6 (full CSA-based rice system), 7 species were eliminated and the density of *P. minor and A. arvensis* was reduced to be less than one plant m^−2^ instead of 25.93 ± 2.69 and 2.33 ± 0.73 (in the first year), respectively. However, the density of *S. nigrum* was markedly increased under all CA/CSA scenarios (Fig. 2). For example, Sc3 recorded 6.0, 4.27, and 2.73-times higher density of *S. nigrum* compared to Sc1 after 1, 4 and 8 years, respectively.

Maximum total weed density (TWD; no m^−2^) was recorded in the first year (Table 2). During 2016–2017 and 2019–2020, all CA/CSA-based scenarios significantly reduced TWD, with Sc5 and Sc6 having the highest suppression effect. Minimum TWD was observed in 2019–2020 in which the TWD was lower than Sc1 by 39.17, 39.72, 53.8, 65.0 and 71.7% for Sc2, Sc3, Sc4 Sc5 and Sc6, respectively. The effect of CSA-based management practices on TWD was cumulative and was maximum in the long-term. For example, following Sc2, TWD was reduced by 11.72, 28.47 and 39.17 after 1, 4 and 8 years, respectively. Similarly, TWD in Sc4 was lower than Sc1 by 5.92, 39.97 and 53.83 after 1, 4 and 8 years, respectively.

**Table 2:** Total weed density and diversity indices (Shannon, Richness and Evenness) under climate-smart agriculture-based management practices during the different years.

Scenario*	Total weed density (no. m^−2^)			Shannon *H′*			Richness Dmg			Evenness *E*		
	2012–2013	2016–2017	2019–2020	2012–2013	2016–2017	2019–2020	2012–2013	2016–2017	2019–2020	2012–2013	2016–2017	2019–2020
Sc1	59.10^Aa^	53.97^A^	49.60^A^	0.34^A^	0.30^A^	0.29^A^	0.19^B^	0.21^C^	0.22^C^	0.028^A^	0.025^A^	0.024^A^
Sc2	52.17^A^	38.60^B^	30.17^B^	0.30^A^	0.32^A^	0.33^A^	0.21^B^	0.30^B^	0.37^BC^	0.025^A^	0.026^A^	0.027^A^
Sc3	43.67^B^	35.13^B^	30.97^B^	0.33^A^	0.28^A^	0.31^A^	0.25^A^	0.32^B^	0.36^BC^	0.027^A^	0.024^A^	0.026^A^
Sc4	55.67^A^	33.03^BC^	22.90^C^	0.31^A^	0.34^A^	0.36^A^	0.20^B^	0.34^B^	0.48^B^	0.026^A^	0.028^A^	0.030^A^
Sc5	NA	32.07^BC^	17.35^D^	NA	0.34^A^	0.33^A^	NA	0.35^B^	0.67^A^	NA	0.028^A^	0.028^A^
Sc6	NA	25.13^C^	14.00^D^	NA	0.33^A^	0.34^A^	NA	0.45^A^	0.79^A^	NA-	0.028^A^	0.028^A^

^*^Refer Table 1 for scenario description. ^a^Different capital letters indicate the means that are significantly different among treatments (least significant difference; P < 0.05).

### Weed biomass

**Figure 3:**
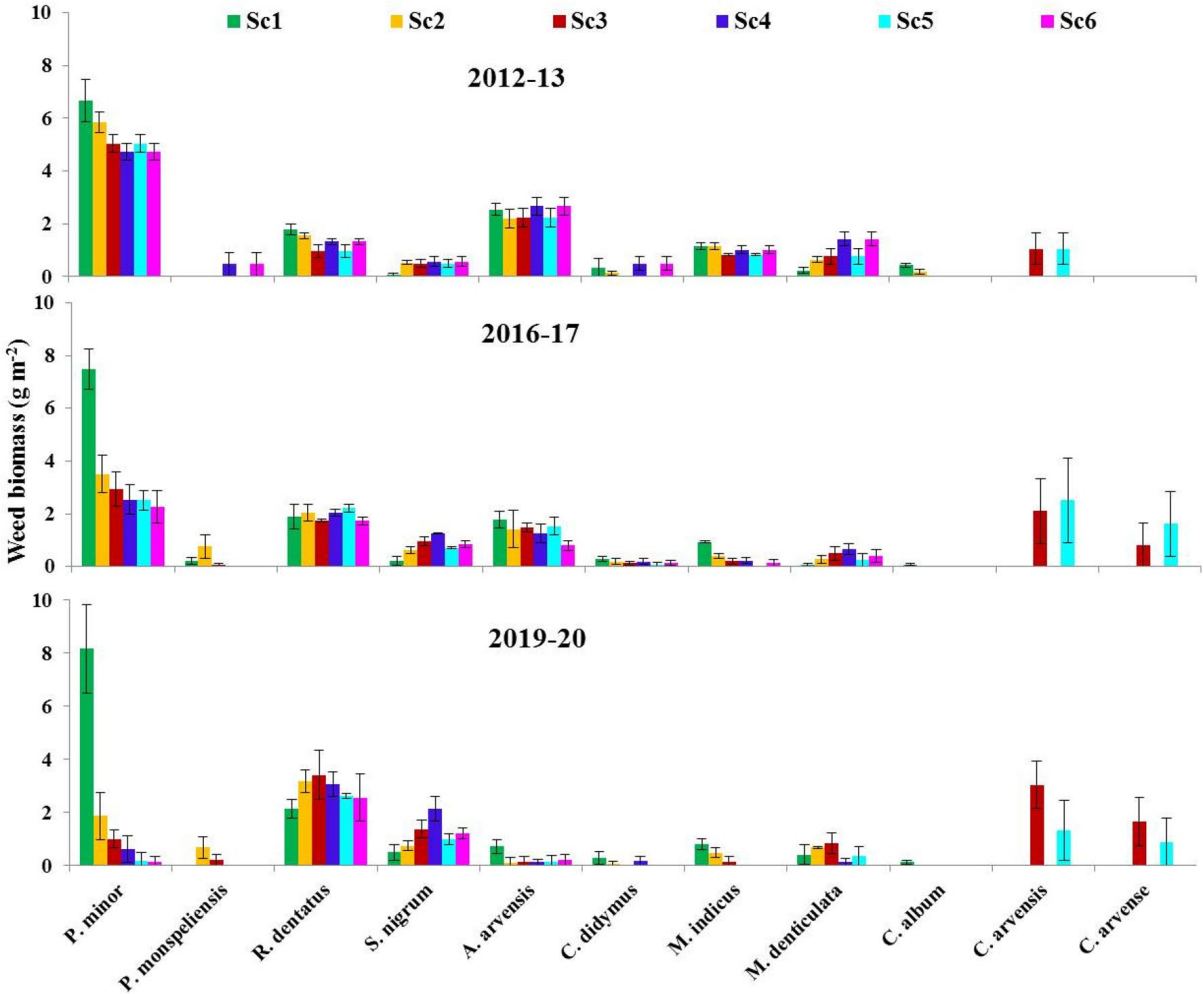
Effects of climate-smart agriculture-based management practices on major weed biomass (g m^−2^) at 45 days after planting during different years. Vertical bars indicate ± SE of mean. *Sc1* conventional rice–wheat system with flood irrigation (FI), *Sc2* conservation agriculture (CA)-based rice–wheat–mungbean system with FI, *Sc3* partial CSA-based rice–wheat–mungbean system with FI, *Sc4* partial CSA-based maize–wheat–mungbean system with FI, *Sc5* full CSA-based rice–wheat–mungbean system with subsurface drip irrigation (SDI), *Sc6* full CSA-based maize–wheat–mungbean system with SDI. The figure has been generate using Microsoft Excell office professional Plus (2010), version (14.0.4734.1000).

Results presented in Fig. 3 showed that weed biomass (dry weight, g m^−2^) was affected by CSA-based management practices and the effect varied between years and species. Initially (in 2012–2013), maximum weed biomass was observed for *P. minor* followed by *A. arvensis, M. indicus, R. dentatus and C. didymus.* Overall, CSA-based management practices distinctly reduced the biomass of four abounded weed species (*P. minor, A. arvensis, M. indicus, and C. didymus*)*,* with full CSA-practices (Sc5 and Sc6) having the greatest effect (Fig. 3). However*,* the biomass of *S. nigrum and M. denticulata* was increased and having the greatest values with partial CSA-practices (Sc3 and Sc4).

The effect of CSA-based management practices on weed biomass was more evident on the long-term, for example, when adopting Sc4, the biomass of *P. minor* was lower than Sc1 by 29.3, 66.2, 92.4% after 1, 4 and 8 years, respectively (Fig. 3). Likewise, under Sc5, the biomass of *P. minor* was lower than Sc1 by 66.3 and 97.8% after 1 and 4 years, respectively. Additionally, Sc6 reduced the biomass of *C. didymus* by 53.3 and 100.0% after 1 and 4 years*,* respectively (Fig. 3). Under Sc4, total weed biomass (TWB) was less than Sc1 by 2.81, 26.13 and 53.78% after 1, 4 and 8 years, respectively (Fig. 3). The corresponding value for Sc2, were 7.23, 25.72 and 38.75%. After 8 years of conversion to CA, maximum TWB was recorded for Sc1 (13 g m^−2^) followed by Sc3 (11.44 g m^−2^). However, minimum TWB was observed in the order of Sc6 (3.79 g m^−2^) < Sc4 (5.75 g m^−2^) < Sc5 (6.25 g m^−2^) < Sc2 (7.62 g m^−2^). During 2020 (after 8 years), the TWB was reduced by 41.38, 12.00, 53.78, 49.76 and 69.53% for Sc2, Sc3, Sc4, Sc5 and Sc6, respectively.

### Diversity indices (Shannon, Richness and Evenness)

The Shannon Diversity Index and Evenness showed that different management scenarios had a non-significant effect on weeds diversity across years (Table 2). However, when considering the richness of species (Richness Dmg), CSA-based scenarios demonstrated a significant effect across years (Table 2). In 2012–2013 (after 1 year), a non-significant effect was recorded due to the management scenarios, the only exception is being Sc3, in which higher Richness was recorded. In the year 2019–2020 (after 8 years), all CSA-based scenarios designated a remarkable effect as compared to Sc1, where a significantly higher Richness Dmg was observed, with Sc5 and Sc6 having the lowest diversity.

## Abundant weed species across the years.

### Phalaris minor

**Figure 4:**
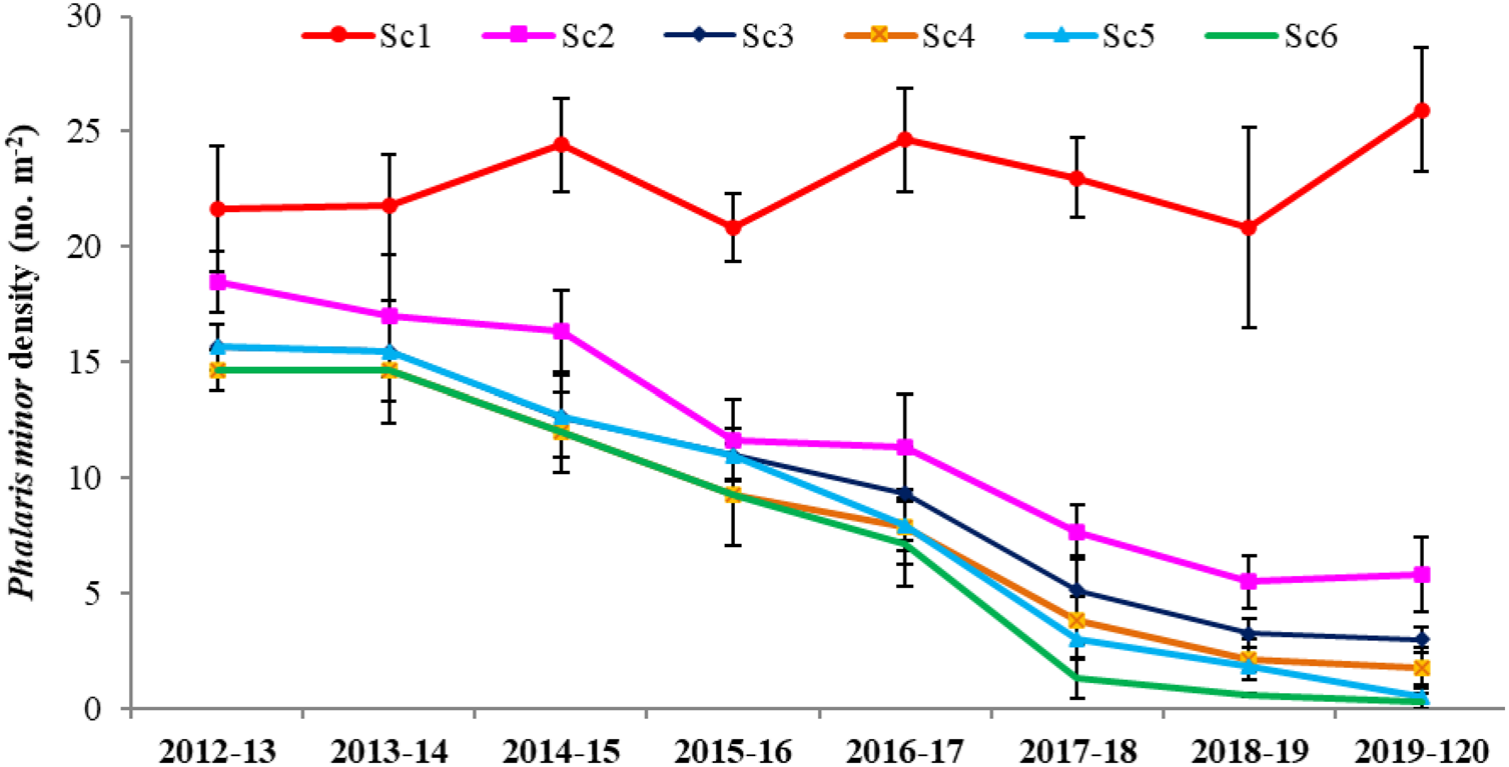
*Phalaris minor* density (no. m^−2^) influenced by climate-smart agriculture-based management practices under wheat crop. Vertical bars indicate ± SE of mean. *Sc1* conventional rice–wheat system with flood irrigation (FI), *Sc2* conservation agriculture (CA)-based rice–wheat–mungbean system with FI, *Sc3* partial CSA-based rice–wheat–mungbean system with FI, *Sc4* partial CSA-based maize–wheat–mungbean system with FI, *Sc5* full CSA-based rice–wheat–mungbean system with subsurface drip irrigation (SDI), *Sc6* full CSA-based maize–wheat–mungbean system with SDI. The figure has been generate using Microsoft Excell office professional Plus (2010), version (14.0.4734.1000).

In general, under Sc1 the density of *P. minor* showed almost the same density across years (2012–2013 to 2019–2020) (Fig. 4). While a downward trend was observed owing to all CSA-based management practices over time. *P. minor* was favored under Sc1, while after 3 years (in 2014–2015), all CSA-based scenarios significantly suppressed its density (with no significant differences among scenarios). Similar trends were observed in 2015–2016 (Fig. 4). After which, (after 4 years), the density of *P. minor* was evidently reduced due to all CA/CSA-based practices, with Sc5 and Sc6 having the utmost effect.

### Rumex dentatus

**Figure 5:**
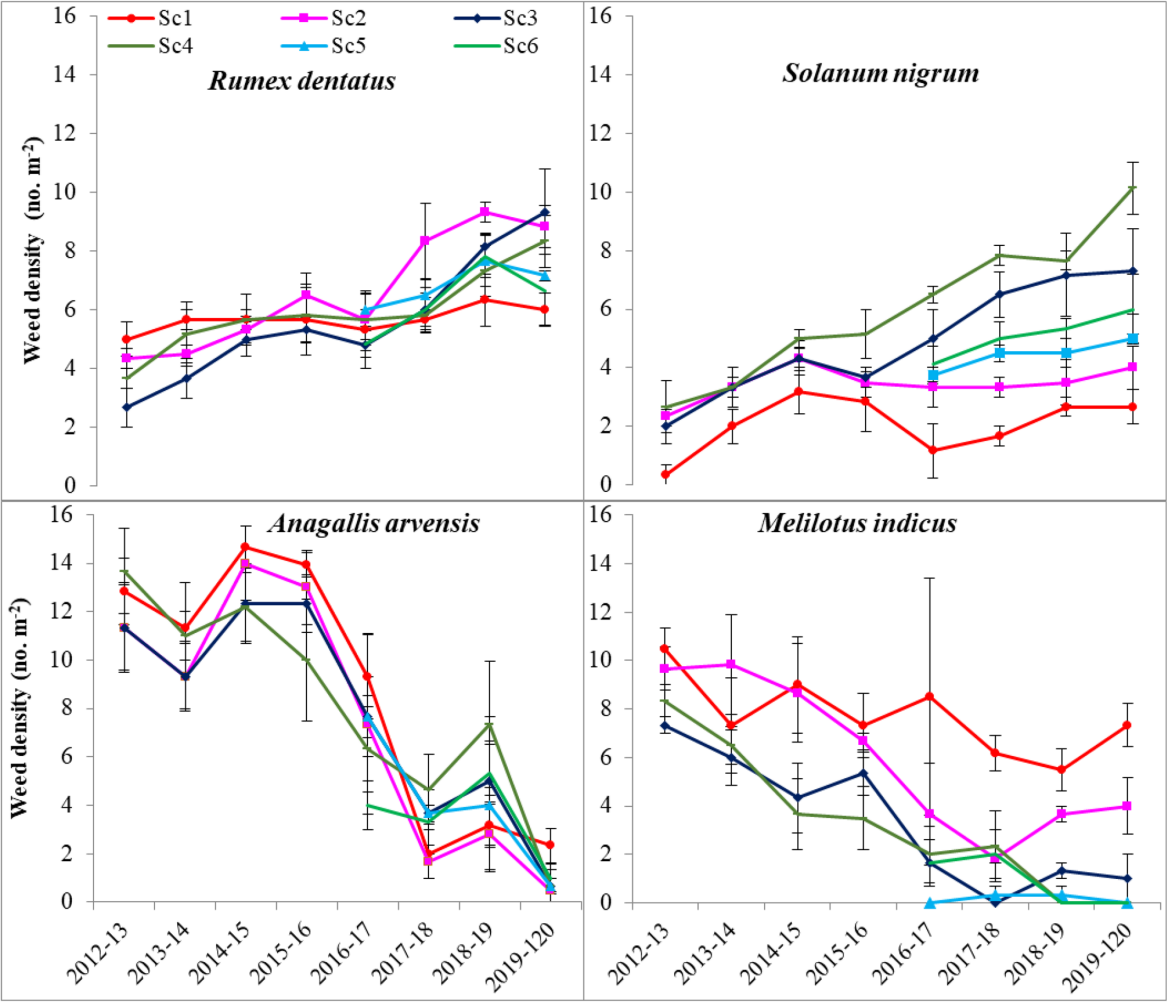
Density (no. m^−2^) of *Rumex dentatus, Solanum. nigrum, Anagallis arvensis* and *Melilotus indicus* as influenced by climate smart agriculture-based management practices under wheat crop. Vertical bars indicate ± SE of mean. *Sc1* conventional rice–wheat system with flood irrigation (FI), *Sc2* conservation agriculture (CA)-based rice–wheat–mungbean system with FI, *Sc3* partial CSA-based rice–wheat–mungbean system with FI, *Sc4* partial CSA-based maize–wheat–mungbean system with FI, *Sc5* full CSA-based rice–wheat–mungbean system with subsurface drip irrigation (SDI), *Sc6* full CSA-based maize–wheat–mungbean system with SDI. The figure has been generate using Microsoft Excell office professional Plus (2010), version (14.0.4734.1000).

The density of *R. dentatus* under Sc1 found to be almost constant (ranged from 5 to 6 plants m^−2^) during the 8 years of study (Fig. 5). However, the density was non-significantly differed from Sc1 in the first 4 years (from 2012–2013 to 2016–2017), except for Sc3 where lower density was observed in the first 2 years (2012–2013 and 2014–2015). After 5 years (from 2017–2018 to 2019–2020), *R. dentatus was* favored by CSA management practices compared to farmers’ practice (Fig. 5).

### Solanum nigrum

In general CSA-based management practices significantly increased the density of *S. nigrum* and was found to be abundant after 8 years of conversion to CSA (Fig. 5). Interestingly, the density of *S. nigrum* was maximum under the maize-based partial CSA system (Sc4)*.* In 2019–2020 (after 8 years), its density was increased by 49.8, 173.4, 229.5, 78.26 and 124.7% over Sc1 for Sc2, Sc3, Sc4, Sc5 and Sc6, respectively.

### Melilotus indicus

The CSA-based management practices gradually and significantly reduced *M. indicus* density across years compared to Sc1 (Fig. 5). When CSAPs have been adopted, the density of *M. indicus* was significantly reduced for all CSA-based scenarios (in the first 4 years). The suppression effect was at greatest level in the last 4 years (2016–2017–2019–2020), where the density ranged from (0.0–1.0 plant m^−2^) under CSA-management practices (Sc3, Sc4, Sc5 and Sc6) instead of 7.33 plant m^−2^ for farmers’ practice (Sc1).

### Anagallis arvensis

The density of *A. arvensis* showed a downward trend along the study period for all CSA-based management practices (Fig. 5). When *A. arvensis,* abundance was naturally high (Sc1), (from 2012–2013 to 2016–2017), all CSA-scenarios non-significantly reduced its density. Moreover, during 2017–2018 and 2018–2019, CSA-scenarios had no effect on the density. However, in the last year (2019–2020), all CSA-scenarios reduced its density by 78.5, 71.2, 71.2, 71.2 and 57.08 for Sc2, Sc3, Sc4, Sc5, and Sc6, respectively.

### Total broadleaf weed density

**Figure 6:**
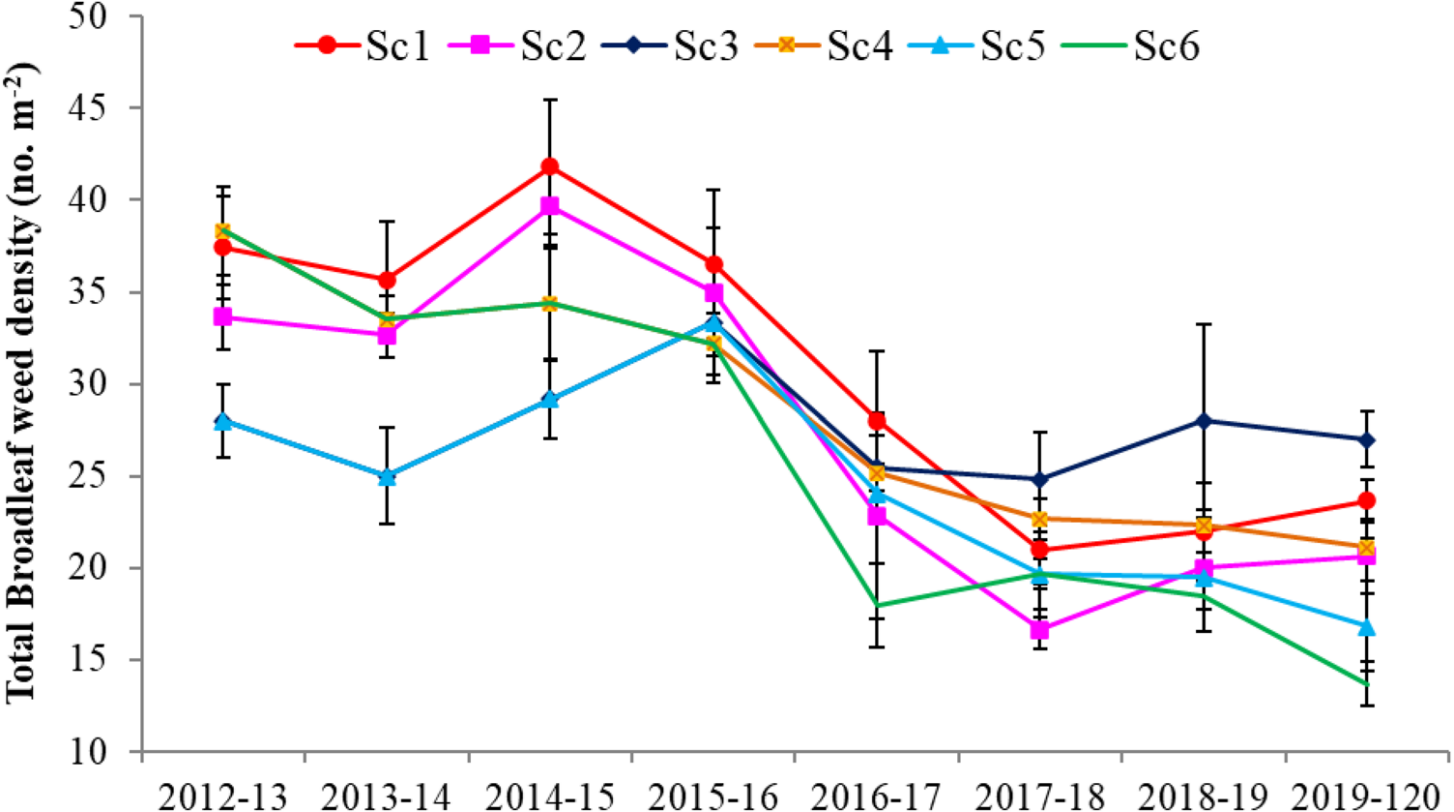
Total broadleaf weed density (no. m^−2^) trend influenced by climate-smart agriculture—based management practices under wheat crop. Vertical bars indicate ± SE of the mean. *Sc1* conventional rice–wheat system with flood irrigation (FI), *Sc2* conservation agriculture (CA)- based rice–wheat–mungbean system with FI, *Sc3* partial CSA-based rice–wheat–mungbean system with FI, *Sc4* partial CSA-based maize–wheat–mungbean system with FI, *Sc5* full CSA-based rice–wheat–mungbean system with subsurface drip irrigation (SDI), *Sc6* full CSA-based maize–wheat–mungbean system with SDI. The figure has been generate using Microsoft Excell office professional Plus (2010), version (14.0.4734.1000).

CSA-based management practices non-significantly affected the density of broadleaf weed. The exceptions are being for Sc3 only in (2013–2014) and Sc2 only in (2017–2018) in which a significant reduction in total broadleaf density has been observed (Fig. 6). During the last 4 years (2016–2017 to 2019–2020), with adapting SDI in rice/maize-based system (Sc5 and Sc6), in the first 3 years (2017–2019) a numerical reduction in broadleaf density (non-significant) was observed. After which (in 2019–2020), both Sc5 and Sc6 significantly reduced the density of broadleaf weeds by 28.9% and 42.3%, respectively (Fig. 6).

### Relative weed density

**Figure 7:**
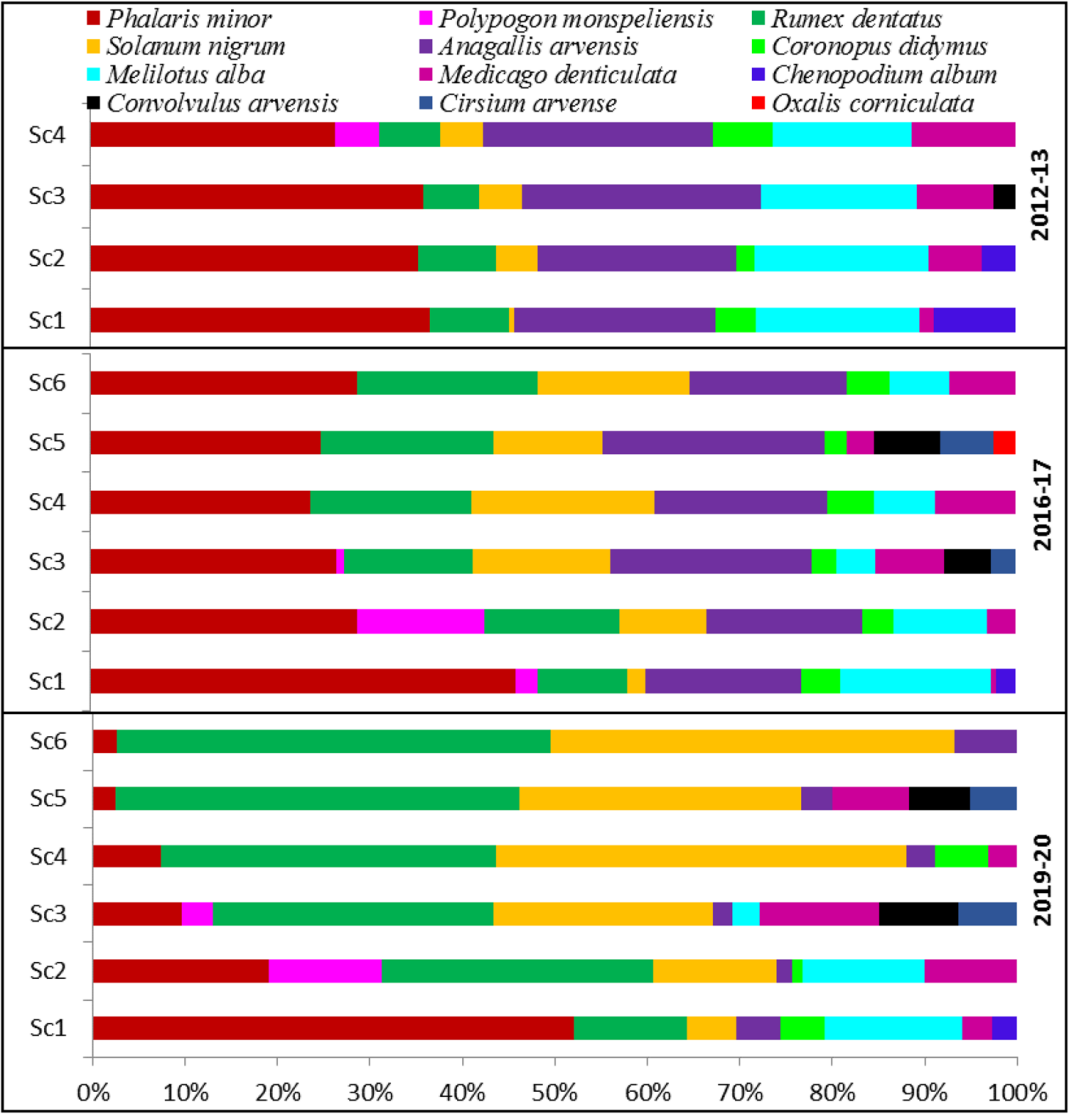
Effects of climate-smart agriculture-based management practices on relative weed density (%) under different scenarios. *Sc1* conventional rice–wheat system with flood irrigation (FI), *Sc2* conservation agriculture (CA)-based rice–wheat–mungbean system with FI, *Sc3* partial CSA-based rice–wheat–mungbean system with FI, *Sc4* partial CSA-based maize–wheat–mungbean system with FI, *Sc5* full CSA-based rice–wheat–mungbean system with subsurface drip irrigation (SDI), *Sc6* full CSA-based maize–wheat–mungbean system with SDI. The figure has been generate using Microsoft Excell office professional Plus (2010), version (14.0.4734.1000).

Relative weed density was affected by different crop management practices. Generally, *P. minor*, *A. arvensis, M. indicus, R. dentatus and S. nigrum* recorded the highest relative density (RD) across the years (Fig. 7). In 2012, *P. minor* recorded the highest RD for all CSA-based scenarios (~ 25 to 35%), followed by *A. arvensis* (~ 25%) and *M. indicus* (~ 20%). Similarly, in 2017, *P. minor* recorded the highest RD for all scenarios, in particular Sc1 (~ 45%). CSA-based management practices increased the RD of both *R. dentatus* and *S. nigrum*, at the expense of *P. minor* and *M. indicus.* In 2020, only in Sc1, *P. minor* was the most abounded species, in which its RD exceeded 50%. However, all CSA-based scenarios intensely reduced its RD to be less than 5% in full CSA-based scenarios (Sc5 and Sc6), irrespective of cropping systems. The reduction in the RD of *P. mino*r, was coupled with an increase in the RD of *R. dentatus* and *S. nigrum*, in particular for Sc4–6. In brief, CSA-practices reduced the RD of *P. minor* over time to be less than 5% in Sc5 and Sc6 in 2019–2020, instead of about 35, 45 and 50% after 1, 4, and 8 years for Sc1 (Fig. 7). Since CSA-practices not only suppressed *P. minor* but also favored both *R. dentatus* and *S. nigrum*, in which the RD of both species was maximum (more than 80–90%) in 2019–2020 under CSA, particularly Sc4, Sc5 and Sc6 instead of 5% for *S. nigrum* and 10% for *R. dentatus* in 2012–2013.

### Contrast analysis

The contrast effects of tillage (CT *vs* ZT), irrigation (flood *vs* SDI) and systems (RW *vs* MW system) on *P. minor* were significant (Table S2). Conventional tillage (CT) had 25, 61 and 86% higher *P. minor* population in 2012–2013, 2016–2017 and 2019–2020 than the ZT system, respectively. Whereas conventional irrigation method (flood) had 43.0 and 95.0% higher *P. minor* in 2016–2017 and 2019–2020 compared to SDI, respectively. Cropping systems also significantly influenced the *P. minor* population in 2016–2017 and 2019–2020, but found similar to 2012–2013 in both the systems (Fig. 2; Table S2). Similarly, the contrast effects of tillage (CT *vs* ZT), irrigation (flood *vs* SDI) and systems (RW *vs* MW system) on broadleaf weed density were significant (Table S2). CT had 11.0, 13.0 and 3.0% higher broadleaf weed density in 2012–2013, 2016–2017 and 2019–2020 than ZT system, respectively whereas the conventional irrigation method (flood) had 17.0 and 34.0% higher broadleaf weed density in 2016–2017 and 2019–2020 compared to SDI, respectively. Cropping systems resulted in a significant influence on broadleaf weed density in 2016–2017 and 2019–2020 but were found similar to the year 2012–2013 in both the systems (Figs. 4, 5; Table S2). Overall, the TWD was also significantly influenced by tillage (CT *vs* ZT), irrigation (flood *vs* SDI) and systems (RW *vs* MW system) (Table 1, Table S2).

## Discussion

Weed abundance was described by determining the density, biomass, and relative density of individual species. In the farmer’s practices (Sc1), weed density, biomass and diversity almost remained the same across the years (2012–2013 to 2019–2020), therefore the observed shift in weed composition was attributed to CSA-based management practices, e.g., tillage, crop residues mulch, cropping system, precision water and nutrients management and layering of these practices. Therefore, the effects of CSA-management practices on weed diversity and density will be discussed as per the influencing drivers/factors.

### Tillage

The observed lower infestation of major weeds under ZT might be attributed to seedbank depletion over time. In ZT, weed seeds accumulate (60–90%) near the soil surface and are more vulnerable to predation, e.g., insects “especially ants”, rodents, birds and other organisms^40^. The higher mortality and loss viability, due to dryness and severe weather variability, lead to more unviable seeds and seedbank depletion over time under ZT^21,25^. The increased number, diversity, and activity of seed-consuming organisms, observed under ZT-fields^41^ complement this opinion. In the current study, the long-term adaption of ZT might contributed to exhausting or depleting seedbank in the soil. In a review article, Nichols et al.^21^ concluded that seedbanks rapidly decreases under ZT than CT. Thus, seedbank could be exhausted after 5 years of implementing ZT^25^. Moreover, in tilled soils weed seedlings emerge from deeper soil layers^42^, which makes the emerged weeds weaker. Similar to our results, several studies have shown that ZT had lower weed infestation of major weeds than CT^25,43^. However, several reports showed higher weed density in ZT with respect to CT even after several years^14,22^, that indicating that other management aspects together with ZT are important.

### Crop residue management

Mulching crop residues might be a compelling reason for the observed reduction in weed density and biomass. In the RW system, retaining crop residues on the soil surface can help in weeds suppression by imposing a physical barrier to emerging seeds, restricting the growth of germinated ones by limiting light availability^44–46^ and by potential allelopathic effects^28,47^. Additionally, surface residue decreases and buffer soil temperature, thus the germination of some weed species’ is reduced due to the reduced fluctuations in soil temperature^48^. Under limited light availability conditions, emerged seedlings become etiolated and weak as germinated seeds search for light^49^. In the IGP, India, in RW cropping system (under ZT) mulching rice residue of 5 t ha^−1^ promoted predation of RW weeds, including *P. minor* and decreased weed density up to 76%^29^. Similarly, retaining rice crop residues of 5.0 and 7.5 t ha^−1^ reduced the total weed biomass by 23.4–30.3 and 35.5–44.1%, respectively^10^. In the current study, the average rice/maize residue load (2012–2012 to 2019–2020) was 9.69, 6.97, 8.50, 7.14 and 8.57 for Sc2, Sc3, Sc4, Sc5 and Sc6 respectively, implying that the rice and maize residues were enough to help in weed suppression. Rice and wheat residues have been identified as exhibiting genetically controlled allelopathy which could be browbeaten for weed suppression^50^. Interestingly, since ZT accumulate seeds close to the soil surface, the allelopathy effect of crop residue (on soil surface) is more suppressive than incorporated residue^47^.

### Efficient crop rotation

RW cropping system is the main cropping system, in the IGP-India, therefore weed is a major problem^29^. In the present study, the inclusion of mungbean to the RW cropping system (all tested CA/CSA-scenarios) might contribute to weed suppression. Similar results were reported by Shahzad et al.^28^ and Malik et al.^46^ in which they found that the inclusion of sorghum, in the wheat-based cropping system may help managing weeds. In the present study, the RW cropping system had the maximum weed diversity, TWD and TWB across years, while maize-based cropping (Sc4 and Sc6), not only showed the least density and biomass, but also had the minimum weeds diversity (Fig. 2), confirming the findings of a review article^21^ and a meta-analysis^30^ which reported that crop rotation distinctly reduces weed densities. Each crop smears certain restrictions on weed flora, which promote the growth of some weeds and suppress others^28,51^. Rotating crops prevents one weed from being repeatedly successful, thus avoiding its establishment^9,31^. Consequently, each crop can act as a filter, allowing only weeds that can adapt with this sort of crop management^31,51^. In the current study, the RW cropping system favored *P. minor* and *A. arvensis* (Figs. 2, 3). However, adapting CSA-based management practices; shifted weed flora toward more broadleaf (e.g., *S. nigrum and R. dentatus*) in all cropping systems and perennial weeds (*C. arvensis* and *C. arvense*) only in ZT-rice-based cropping system. The presence of perennial weeds in CA has been previously reported^9,21^. Interestingly, using different crops also increases predation pressure^33,52^ and promote predation of distinct weed seeds^34^. Therefore, rotating crops could increase the diversity of consumed seeds, thus reduce intensification.

### Precise water management

Precise water management (through subsurface drip irrigation; SDI) is a promising technology for altering weed density and weed suppression effects^53^. The positive effect of SDI might be due to adjusting water and fertilizer placement. Since under ZT weed seeds accumulate near and/or on the soil surface, weeds germination is dramatically reduced because SDI leaves the few top cms of the soil dry^54,55^. Additionally, because wheat can germinate in drier environments than can various weeds^56^, sowing under drier conditions (SDI) can reduce weed emergence especially, moisture-loving weeds like *P. minor*^57^. Interestingly, placement of water and fertilizer using SDI can discourage weed growth rates^21,58^, as SDI better deliver water and nutrients where it will most benefit crops, not weeds^53,55^. Shrestha et al.^54^ found that weed abundance under SDI almost vanished. Similarly, several studies reported that fertilizers’ deep banding might decrease weed biomass compared to surface banding or broadcasting^53,59^.

### Weed density and diversity under CSA-based management

The CSA-based management practices suppressed weed communities and shifted their composition. Under conventionally managed RW system, *P. minor, A. arvensis, M. indicus and C. album* were favored, while CSA practices favored *S. nigrum* and *R. dentatus*. Therefore, *P. minor, A. arvensis, M. indicus, C. album*, *S. nigrum* and *R. dentatus* are the best indicators for the crop management practices in the IGP of south Asia. The lower emergence of *P. minor* under CSA-based management practices might be due to surface soil strength in ZT (after rice harvest) which obstruct weeds emergence, higher predation of weed seeds and low light availability; all of which weaken germination of many weed species not only *P. minor*^60^. On the other side, the higher density and biomass of both *S. nigrum* and *R. dentatus,* under all CA/CSA-scenarios, might be due to the vast majority of their seeds accumulate near to the soil surface where they can better germinate^10,14^. In contrast, under CT, during tillage, seeds of *S. nigrum* and *R. dentatus* are buried and their emergence is markedly decreased due to their high sensitivity to seeding depth^14,61^. The germination of *S. nigrum* was 93.1 and 4.7% at a seeding depth of 1 and 4 cm, respectively, while its emergence was inhibited at 8 cm seeding depth^61^. In the present study, the effect of CSA-based management practices are additive. Using the same tillage practices (ZT), crop residue retention and cropping system, but with different water/nutrients application (flooding vs SDI), led to more significant weed suppression. Precise water management (through SDI) is promising CSA-practice in weed suppression and showed the superiority of Sc6 over Sc4 and Sc5 over Sc3.

The combined-additive effect of CSA-practices on weed flora composition and infestation levels was more evident in the long-term. Similar to our observations, a non-significant difference in total weed density (TWD) and TW biomass (B) in the first year was observed by other researchers^22,62^. After 4 years, a significant reduction in both TWD and TWB was observed. This suppression might be ascribed to weed seedbanks depletion^21,25^. About 4–10 years are required to reach the weed population’s equilibrium^63^ and it was observed in our study as well with respect to the TWD and TWB. The modern planting technology (Combine harvesting with super SMS followed by Happy Seeder sowing in wheat) have made wheat sowing successfully possible under heavy rice residues (8–10 t ha^−1^) and eased the use of residues for weed management without any negative effect on crop establishment^46,64^. In this study, ZT under residues retention conditions and integrated with pulse crop (mungbean) and SDI would evidently deplete the weed seedbank.

The collective use of all CSA practices could offer additive benefits, and the potential of weed infestation is higher if only one management practice is applied. Our results imply that layering of CSA-based crop management practices; ZT, crop rotation, crop residue retention and SDI are synergistic means of weed control. Minimum TWD was observed for Sc6, followed by Sc5 and Sc4. Indicating the importance of SDI as CSA practices to minimize weed effects. In terms of TWB, Sc6 recorded minimum TWB, followed by Sc4 and Sc5, implying the importance of the cropping system (replacing rice with maize). This might be due to that weed density does not always represent weed biomass (more feasible when emphasizing crop-weed competition)^21^. The superiority of CSA-based maize systems could be a promising alternative for the RW cropping system, where less weed diversity, density and biomass were recorded. This promotes a big opportunity for weed suppression in IGP, south Asia.

## Conclusion

Crop management activities like tillage, crop residue, crop rotations, water application and nutrients management affect weed diversity and composition. Weed community responses to long-term climate-smart agriculture (CSA) management practices in cereal-based agri-food systems of western IGP was investigated. The most abundant species were *P. minor, A. arvensis, M. indicus* and *C. album* and were favored by farmers’ practice. However, CSA-based management practices markedly reduced total weeds density and biomass and shifted weed flora towards broadleaf weed species i.e., *S. nigrum* and *R. dentatus*. The effect of CSA-based management practices on weed flora composition and infestation levels was additive and more evident in the long-term. Implementing long-term CSA-practices might lead to weed seedbank depletion due to encouraging weed predation factors. The superiority of Sc4 over Sc3 and Sc6 over Sc5 “in weed suppression” implies the importance of crop rotation (replacing rice with maize). Similarly, the superiority of Sc6 over Sc4 and Sc5 over Sc3 signifying the role of subsurface drip irrigation (SDI) is promising CSA practice in weed suppression. Our results indicate that layering of CSA management practices was found to be synergistic means of weed control. In conclusion, full CSA-based maize–wheat–mungbean system could be a promising alternative for the rice–wheat system for better weed management in western IGP. Besides the measurement of weed density and biomass, in long-term experiments future studies should also focus on changes of seedbank across years to get more in-depth clarity on weed behavior and dynamics.

## Materials and methods

### Site description

A study was initiated in 2012–2013 at ICAR-Central Soil Salinity Research Institute, Karnal, (29° 70′ N, 76° 95′ E), India to understand the shift in weed flora in cereal-based cropping systems under different climate-smart agriculture (CSA)-based management practices. During the rainy season, rice and maize crops were grown followed by wheat in the winter season as per the treatment protocols. The site has a semi-arid and sub-tropical climate, with hot and dry to wet summers and cold dry winters. The average annual rainfall (40-year period) was 765 mm with mean maximum temperature was 37.7 °C in June, whereas the minimum temperature was 6.4 °C in January. The soil of the experimental field is silty loam in texture, low in organic carbon (0.45%) with neutral pH. Before conducting this experiment, rice–wheat (RW) cropping system was being practiced under conventional tillage (CT)-based management system for 30 years.

### Experimental design and treatment details

The experiment was started (2009–2010) with four cereal-based scenarios (Sc) that differ in tillage and crop establishment, cropping system, crop residue management, and other crop management practices under the CSISA (Cereal Systems Initiative for South Asia) project to address the sustainability issues of RW system in the western IGP. The observation on weed density and composition was started in 2012–2013. Initially, the experiment comprised four cereal-based scenarios (Sc1–Sc4) varied with different range of indicators in a plot scale (20 m × 100 m) and based on the conservation agriculture (CA) principles. In May 2016, Sc3 and Sc4 plots were subdivided (plot size: 20 m × 50 m) and one subdivided plot was layered with precise water and N-management through subsurface drip irrigation (SDI) across all the replications and designated as Sc5 and Sc6, respectively. Briefly, six scenario (Sc) comprised were: (1) conventional-till (CT) rice–CT wheat (Sc1; farmers’ practice; CT); (2) CT rice-Zero tillage (ZT) wheat–ZT mungbean with flood irrigation (Sc2; partial CA-based rice system); (3) ZT rice–ZT wheat–ZT mungbean with flood irrigation (Sc3; partial CSA-based rice system); (4) ZT maize–ZT wheat–ZT mungbean with flood irrigation (Sc4; partial CSA-based maize system); (5) ZT rice–ZT wheat–ZT mungbean with SDI (Sc5; full CSA-based rice system); and (6) ZT maize–ZT wheat–ZT mungbean with SDI (Sc6; full CSA-based maize system). The scenarios were arranged in a complete randomized block design, using three replicates. Details of the tested scenarios including drivers of change, crop rotations, tillage, crop establishment method, and residue and water management practices are given in Table 1. Basically, Sc3 and Sc4 were based on CA practices, in which irrigation water and N application were not precisely managed and called it partial climate-smart agriculture (CSA). However, in Sc5 and Sc6, irrigation water and N- was precisely applied SDI and called full CSA. In all scenarios, best crop management practices were applied except Sc1, in which the traditional practices of farmers were followed (Table 1). We used four systems for better understanding, conventional rice–wheat system (Sc1), partial CA-based rice–wheat–mungbean system (Sc2), partial CSA-based rice/maize system (mean of Sc3 and Sc4) and full CSA-based rice/maize system (mean of Sc5 and Sc6). The CSA-based systems include the Sc3 and Sc5 for rice and Sc4 and Sc6 for maize systems.

### Crop residue management

In the farmers’ practice (Sc1) all the crop residues were removed from the ground level. In rice-based systems (Sc2, Sc3 and Sc5), full (100%) residue of rice was retained on the soil surface in wheat crop. However, in maize-based systems (Sc4 and Sc6), full (100%) resides of maize were retained for the first 3 years and for the remaining years partial (65%) maize residue was retained in the wheat crop. The incorporation or retention of the crop residue was depended on the biomass production of the previous crops that varied from 1 year to another years. In different scenarios, the total amount of rice crop residue ranged from 5.30 to 12.70 Mg ha^−1^, while for maize it was 5.80–13.10 Mg ha^−1^ in 8 years of study (Table S3). The average annual crop residue load was ranged from 6.97 to 9.69 Mg ha^−1^ across the different management scenarios (Table S3).

### Crop management

In Sc1, both rice and wheat were conventionally established following farmers’ practice. In CT rice plots, dry-tillage (two harrowing and two cultivators followed by wooden planking), and wet-tillage (puddling; two harrowing and one planking) were done before rice transplanting. In CT wheat, two passes of harrowing and cultivator each followed by planking was done prior to sowing. Under Sc1, rice was manually transplanted in puddled fields with 25–30 days old seedlings, while wheat was planted by manual broadcasting in tilled soil. In Sc2, after wet-tillage (three passes of harrowing and one planking in standing mungbean crop after picking), rice was manually transplanted in a random geometry (20 × 15 cm). Under ZT conditions (Sc3–Sc6) rice, wheat and mungbean were planted in rows (22.5 cm apart) using Happy Seeder with an inclined plate seed metering mechanism. However, maize was seeded by Happy Seeder at a row spacing of 67.5 cm. Rice (hybrid Arize 6129) was seeded with a seed rate of 10 and 20 kg ha^−1^ in CT and ZT plots, respectively. However, wheat (HD 2967) was seeded at a seed rate of 120 kg ha^−1^ in CT plots and 100 kg ha^−1^ in ZT plots. Both maize (hybrid, DKC 9125) and mungbean (cultivar, SML 668) were seeded at a seed rate of 20 kg ha^−1^. For disease tolerance, seeds of all crops except mungbean were treated (prior to seeding), with fungicides, tabuconazole (Raxil 60 FS) (1 ml kg^−1^ seed) and imidachloropid (Guicho 600 FS) (5 g kg^−1^ seed). During the last week of October *(Rabi* season), wheat was sown, while rice and maize were seeded in the *Kharif* season. Every year, short-duration mungbean (from mid-April to mid-June) was grown between wheat harvest and rice sowing. Under Sc1, 175 + 00 + 00 kg NPK ha^−1^ were applied in the wheat crop as per the farmers’ practice, whereas, in Sc2–Sc4, 150 + 60 + 60 kg NPK ha^−1^ were applied. Under subsurface drip fertigation plots (Sc5 and Sc6), the recommended rate of N-fertilizer (150 kg N ha^−1^) was decreased by 20%. In SDI, four equal doses of N through fertigation at 25, 45, 65 and 85 DAS were applied in wheat crop, whereas with flood irrigation N was applied in two equal doses at 1st and 2nd irrigation.

### Weed management

Before seeding, no herbicides were applied in conventional-till (CT) plots i.e., sc1 (farmer’s practice), while glyphosate @ 900 g a.i. ha^−1^ was applied prior to sowing in zero-till (ZT) plots (Sc2–Sc6). To control grassy and broadleaf weeds, a mix solution of clodinafop-ethyl + metsulfuron @60 + 4 g a.i. ha^−1^ or sulfosulfuron + metsulfuron @32 g a.i. ha^−1^ were used from the year 2012–2013. However, from the year 2016–2017 onwards, pinoxaden (5EC)-axil@1000 ml/ha + metsulfuron methyl 20% WG-algrip@20 g a.i. ha^−1^ were used at 35 days after sowing as and when required (Table S4). Before spraying of herbicides in wheat crop, four demarked areas (quadrate of 1.0 m^2^ each) in each plot from where samples were taken (after 45 DAS) was covered with polythene. Every year the sampling location was different to know the real diversity and density of weed species.

### Weed observations

Authors confirm that experiment on weeds/plant species in the present study complies with the Institute guidelines. Weed samples were taken at 45 days after sowing (DAS) of the wheat crop, when most of the weeds emerged. Every year, weeds were identified and counted species wise from four places in each plot/scenario by using a quadrate of 1.0 m^2^. Formal identification of the weed species was done by the first author in consultation with Dr. Virender Kumar, weed scientist. Total weed density (TWD) was calculated by summing the individual density of all weeds and expressed as (no. m^−2^). Similarly, the density of grassy and broadleaf weeds (no. m^−2^) was counted by summing the individual densities of grassy and broadleaf weeds, respectively. Weed biomass (g m^−2^) was determined by drying the collected weed samples at 65 °C for 72 h. Twelve weeds (representing grassy and broadleaf weeds) viz. *Phalaris minor* Retz, *Polypogon monspeliensis* (L.) Desf, *Rumex dentatus* L., *Solanum nigrum* L., *Anagallis arvensis* L., *Coronopus didymus* (L.) Sm, *Melilotus indicus* (L.), *Medicago denticulata* Willd, *Chenopodium album* L., *Convolvulus arvensis* L. and *Cirsium arvense* (L.) Scop were observed over the years, while *Oxalis corniculata* found negligible in wheat crop (Table S1). The voucher specimen of the weed species has not been deposited in a publicly herbarium, as there is no available a publicly herbarium.

### Weed diversity indices

Relative weeds density (RD, %) of species in the whole weed community was calculated as the ratio between the density of a given weed specie to total weed density in each scenario. Weed’s biodiversity was measured, including (1) the species richness (S), i.e., the number of species existed in a quadrat; (2) the species diversity, which was calculated using Shannon–Wiener index as ${H}^{^{\prime}}= -\sum Pi\times ln Pi$, where P*i* is the portion of individual numbers of the *i* species to the total individual number of each species in the quadrat. The P*i* was calculated as $Pi=Ni/N$ of which N is the total individual number of each weed species and N*i* is the individual number of the *i* species; (3) the degree of community dominance, which was calculated by the Simpson index as ${D}^{^{\prime}}= \sum P{i}^{2}$; and (4) the community evenness, which was calculated by the evenness index (Pielou index).

### Statistical analysis

The data of weed parameters were analyzed using analysis of variance (ANOVA) according to Gomez and Gomez^65^, for randomized block design using SAS 9.1 software (SAS Institute, 2001). The treatment means were separated using Tukey’s honestly significant difference (HSD) at 5% level of significance. The mean effects of tillage, cropping systems and irrigation methods were determined using linear contrast or individual factor in the JMP. All figures have been generated using Microsoft office professional Plus^66^ version (14.0.4737.1000).

### Ethical approval

Experiment was conducted after taking proper approval from the Institute Research Committee of ICAR-CSSRI, Karnal. Guidelines of the ICAR-CSSRI were followed for taking data of crop/weed/plants.

## Supplementary Information

Supplementary Information 1.
