# Supplementary material for: Climate-smart agriculture practices influence weed density and diversity in cereal-based agri-food systems of western Indo-Gangetic plains
Source: Sci Rep. 2021 Aug 5;11:15901. doi: 10.1038/s41598-021-95445-1 (PMC8342518; doi:10.1038/s41598-021-95445-1)
Supplement: Supplementary file 1 — Supplementary Information 1. [file 41598_2021_95445_MOESM1_ESM.docx]

**Supplementary tables**

**Climate-smart agriculture practices influence weed density and diversity in cereal-based agri-food systems of western Indo-Gangetic plains**

Hanuman S. Jat^a,f*^, Virender Kumar^b^, Suresh K. Kakraliya^a^, Ahmed M. Abdallah^c^, Ashim Datta^a^, Madhu Choudhary^a^, Mahesh K. Gathala^d^, Andrew J. McDonald^e^, Mangi L. Jat^f^, Parbodh C. Sharma^a^

^a^ICAR-Central Soil Salinity Research Institute (CSSRI), Karnal, India

^b^International Rice Research Institute (IRRI), Los Banos, Philippines

^c^Faculty of Agriculture, Damanhour University, Damanhour, Egypt.

^d^International Maize and Wheat Improvement Center (CIMMYT), Dhaka, Bangladesh

^e^Collage of Agriculture and Life Sciences, Cornell University, Ithaca NY USA

^f^International Maize and Wheat Improvement Center (CIMMYT), New Delhi, India

| **S No.** | **Botanical Name** | **Family** | **Common Name** | **Annual/Perennial** |
| --- | --- | --- | --- | --- |
| 1. | *Phalaris minor* Retz | Poaceae | Mandusi, Little seed canary grass | Annual herb- Grass |
| 2. | *Polypogon monspeliensis* (L.) Desf | Poaceae | Rabbit`s foot grass, Lumbad gash | Annual herb- Grass |
| 3. | *Rumex dentatus* L. | Polygonaceae | Jangli palak | Annual or biennial herb- Broad leaf |
| 4. | *Solanum nigrum* L. | Solanaceae | Nightshade, Makoy | Perennial shrub- Broad leaf |
| 5. | *Anagallis arvensis* L. | Primulaceae | Krishan neel, Billi booti, | Perennial- Broad leaf |
| 6. | *Coronopus didymus* (L.) Sm | Brassicaceae | Jangli halon, Pit papra | Annual or biennial herb- Broad leaf |
| 7. | *Melilotus indicus* L. | Fabaceae | Yellow sweet clover | Annual or biennial legume- Broadleaf |
| 8. | *Medicago denticulata* Willd | Fabaceae | Sweet clover**,** Senji | Annual herb- Broad leaf |
| 9. | *Chenopodium album* L. | Chenopodiaceae | Bathua | Annual- Broad leaf |
| 10. | *Convolvulus arvensis* L. | Convolvulaceae | Lehli, Hirankhuri | Perennial herb- Broad leaf |
| 11. | *Cirsium arvense* (L.) Scop | Asteraceae | Canada thistle, Kandai | Perennial herb- Broadleaf |

**Table S1.** Common weed species prevailed under varied CSA- based management scenarios.

| Scenarios | *Phalaris minor* density (No. m^-2^) | | | Broadleaf weed density (No. m^-2^) | | | Total weed density  (No. m^-2^) | | |
| --- | --- | --- | --- | --- | --- | --- | --- | --- | --- |
|  | 2012-13 | 2016-17 | 2019-20 | 2012-13 | 2016-17 | 2019-20 | 2012-13 | 2016-17 | 2019-20 |
| CT *vs* ZT | 0.02 | <0.001 | <0.0001 | 0.15 | 0.13 | 0.036 | 0.01 | 0.0008 | <0.0001 |
| Flood *vs* SDI | - | 0.006 | <0.0001 | - | 0.09 | <0.0001 | - | 0.009 | <0.0001 |
| Cropping system (RW *vs* MW) | 0.07 | 0.006 | <0.0001 | 0.07 | 0.16 | 0.004 | 0.16 | 0.012 | <0.0001 |

**Table S2.** Interaction and contrast analysis between year, climate-smart agriculture scenarios and weed density. *Values below 0.05 are significant.

| Scenarios^a^ | Residue incorporated/retained (Mg ha^-1^) | | | | | | | | |
| --- | --- | --- | --- | --- | --- | --- | --- | --- | --- |
|  | 2012-13 | 2013-14 | 214-15 | 2015-16 | 2016-17 | 2017-18 | 2018-19 | 2019-20 | Average |
| Sc1 | -NA^b^- | -NA- | -NA- | -NA- | -NA- | -NA- | -NA- | -NA- | -NA- |
| Sc2 | 10.6 | 10.6 | 12.7 | 7.93 | 8.25 | 8.35 | 8.35 | 10.73 | 9.69 |
| Sc3 | 5.3 | 5.7 | 6.9 | 6.71 | 7.25 | 7.58 | 7.58 | 8.73 | 6.97 |
| Sc4 | 10 | 10.2 | 13.1 | 5.8 | 6.69 | 6.7 | 6.70 | 8.84 | 8.50 |
| Sc5 | 5.3 | 5.7 | 6.9 | 7.12 | 7.24 | 8.04 | 8.04 | 8.80 | 7.14 |
| Sc6 | 10 | 10.2 | 13.1 | 5.39 | 7.01 | 6.85 | 6.85 | 9.12 | 8.57 |

**Table S3.** Rice/maize crop residue load (Mg ha^-1^) in wheat crop under different scenarios. ^a^Refer to table 1 for scenario description. ^b^Not applicable.

| **Scenarios** | **2012-13** | | | **2016-17** | | | **2019-20** | | |
| --- | --- | --- | --- | --- | --- | --- | --- | --- | --- |
|  | 1^st^ top dress | 2^nd^ top dress | 3^rd^ top dress | 1^st^ top dress | 2^nd^ top dress | 3^rd^ top dress | 1^st^ top dress | 2^nd^ top dress | 3^rd^ top dress |
| Sc1 | BA | BA | SH | BA | BA | SH | BA | BA | SA+SH |
| Sc2 | BA | BA | × | BA | SA | × | BA | SH | × |
| Sc3 | BA | SA | × | BA | SH | × | BA | SH | × |
| Sc4 | BA | SA | × | BA | SH | × | BA | SH | × |
| Sc5 | BA | SA | × | BA | × | × | BA | × | × |
| Sc6 | BA | SA | × | BA | × | × | BA | × | × |

**Table S4.** Herbicides used to control the weeds under different CSA- based management scenarios in different years. BA- blanket application; SA- spot application; SH- spot hand weeding.
